# ^1^H and ^13^C-NMR data for novel meroterpenoids isolated from *Arnebia euchroma* (Royle) Johnst

**DOI:** 10.1016/j.dib.2019.103908

**Published:** 2019-04-09

**Authors:** Yang Wang, Yuzhen Zhu, Lingyun Xiao, Lanlan Ge, Xin Wu, Weigang Wu, Haoqiang Wan, Keda Zhang, Jiemei Li, Boping Zhou, Jun Tian, Xiaobin Zeng

**Affiliations:** a Center Lab of Longhua Branch, Shenzhen People's Hospital, 2nd Clinical Medical College of Jinan University, Shenzhen 518120, China; b Guangdong Key Laboratory for Research and Development of Natural Drugs, Guangdong Medical University, Zhanjiang 524023, China; c Department of Infectious Disease, Shenzhen People's Hospital, 2nd Clinical Medical College of Jinan University, Shenzhen 518120, Guangdong Province, China; d Department of Pathology (Longhua Branch), Shenzhen People's Hospital, 2nd Clinical Medical College of Jinan University, Shenzhen 518120, Guangdong Province, China; e College of Life Science, Jiangsu Normal University, Xuzhou 221116, Jiangsu Province, China; f Key Lab for New Drug Research of TCM and Shenzhen Branch, State R&D Centre for Viro-Biotech, Research Institute of Tsinghua University in Shenzhen, Shenzhen 518057, Guangdong, China

## Abstract

The data presented in this article are associated with the research article entitled *“***Meroterpenoids isolated from *Arnebia euchroma* (Royle) Johnst. and their cytotoxic activity in human hepatocellular carcinoma cells***”* [1]. The aim of this data was to provide the 1D-NMR spectrum of novel meroterpenoids from *Arnebia euchroma* (Royle) Johnst.

**Table:** Specifications table

Subject area	*chemistry*
More specific subject area	*Natural products research*
Type of data	*Figure, Table*
How data was acquired	^*1*^*H and*^*13*^*C-NMR of meroterpenoids*
Data format	*Filtered and Analyzed*
Experimental factors	*First, the Sample were isolated from dichloromethane fraction of the roots of A. Euchroma extracts. Then the samole were dissolved in* DMSO‑*d*_6_*or CD*_*3*_*Cl before NMR test.*
Experimental features	*Nuclear magnetic resonance (NMR) spectra data of meroterpenoids from the roots of A. Euchroma were recorded on a Bruker DPX-400 spectrometer using standard Bruker pulse programs (Bruker, Karlsruhe, Germany). Chemical shifts were shown as δ-values with reference to tetramethylsilane (TMS) as an internal standard.*
Data source location	The herbarium of Key Laboratory for New Drug Research of TCM, Research institute of Tsinghua University in Shenzhen, Shenzhen, China.
Data accessibility	*Data is with this article*
Related research article	Yang Wang, Yuzhen Zhu, Lingyun Xiao, Lanlan Ge, Xin Wu, Weigang Wu, Haoqiang Wan, Keda Zhang, Jiemei L, Boping Zhou, Jun Tian, Xiaobin Zeng. Meroterpenoids isolated from *Arnebia euchroma* (Royle) Johnst. and their cytotoxic activity in human hepatocellular carcinoma cells.Fitoerapia. 131 (2018) 236–244.

**Table:** 

**Value of the data** • NMR data of meroterpenoids is useful for elucidating their chemical structures. • NMR data of meroterpenoids is useful for elucidating their chemical analogues. • This information will allow comparisons across different meroterpinoids from algal species or othernatural sources.

## 1 Data

In our previous study [1], six previously undescribed naturally occurring meroterpenoids (**2**, **5**–**9**) together with seven known meroterpenoids (**1**, **3**, **4**, **10**–**13**) were isolated from the root plant of *Arnebia euchroma*. The NMR data of meroterpenoids **1**–**5** and **8** suggest they were structure analogue, which contain a bridgehead double bond, which has recently attracted substantial interest in the natural product isolation community in terms of Bredt's law. Six previously undescribed naturally occurring meroterpenoids (**2**, **5**–**9**) were first isolated and identified from *Arnebia euchroma* (Royle) Johnst.

## 2 Experimental design, materials, and methods

### 2.1 Study area description

*Arnebia euchroma* (Royle) Jonst. (family Boraginaceae) is a small genus of annual or perennial herbs, distributed in Asia and the drier regions of Northern Africa [2]. *A. Euchroma* is a traditional Chinese herbal medicine (TCM) recorded in the Pharmacopoeia of China and has been extensively used in China and other countries for the treatment of various diseases [3]. In the current study, a further phytochemical investigation on the CHCl_3_ extract of the roots of *A. euchroma* led to the isolation and characterization of six (**2**, **5**–**9**) previously undescribed and seven (**1**, **3**–**4**, **10**–**13**) known meroterpenoids. Herein, their structure characterization of these meroterpenoids are identified by various chromatography methods including NMR and MS spectrum.

### 2.2 Sample collection

The roots of *Arnfebia euchroma* were purchased in Haozhou city, Anhui Province, China.

### *2.3* ^*1*^*H and*^*13*^*C NMR* spectrum *of meroterpenoids (****2****,****5****–****9****)*

The dichloromethane-H_2_O (1: 1, v/v) extract from the roots of *A. Euchroma* was successively subjected to column chromatography over silica gel, ODS or Sephadex LH-20, and preparative HPLC to afford six previously undescribed meroterpenoids (**2**, **5**–**9**) together with seven known meroterpenoids (**1**, **3**–**4**, **10**–**13**). The known compounds were determined to be 9,17-epoxyarnebinol (**1**) [4], arnebinol B (**3**) [5], arnebinone B (**4**) [6], arnebifuranone (**10**) [7], shikonofuran A (**11**) [8], shikonofuran E (**12**) [8], and arnebinone (**13**) [9], by comparison of their spectral data with literature values.

#### 2.3.1 Meroterpenoid **2**

**Fig. 1:**
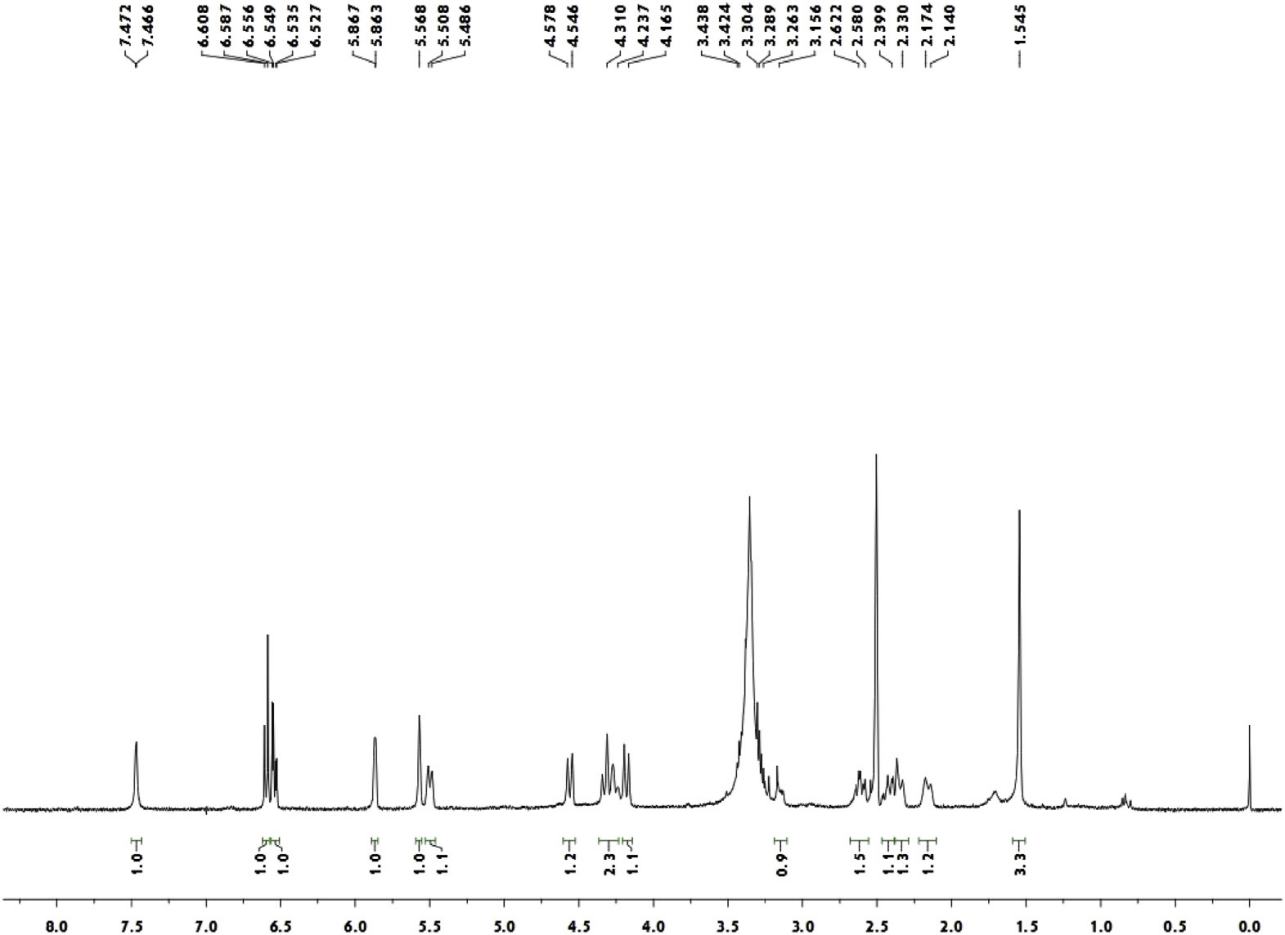
^1^H-NMR spectrum of meroterpenoid **2**.

**Fig. 2:**
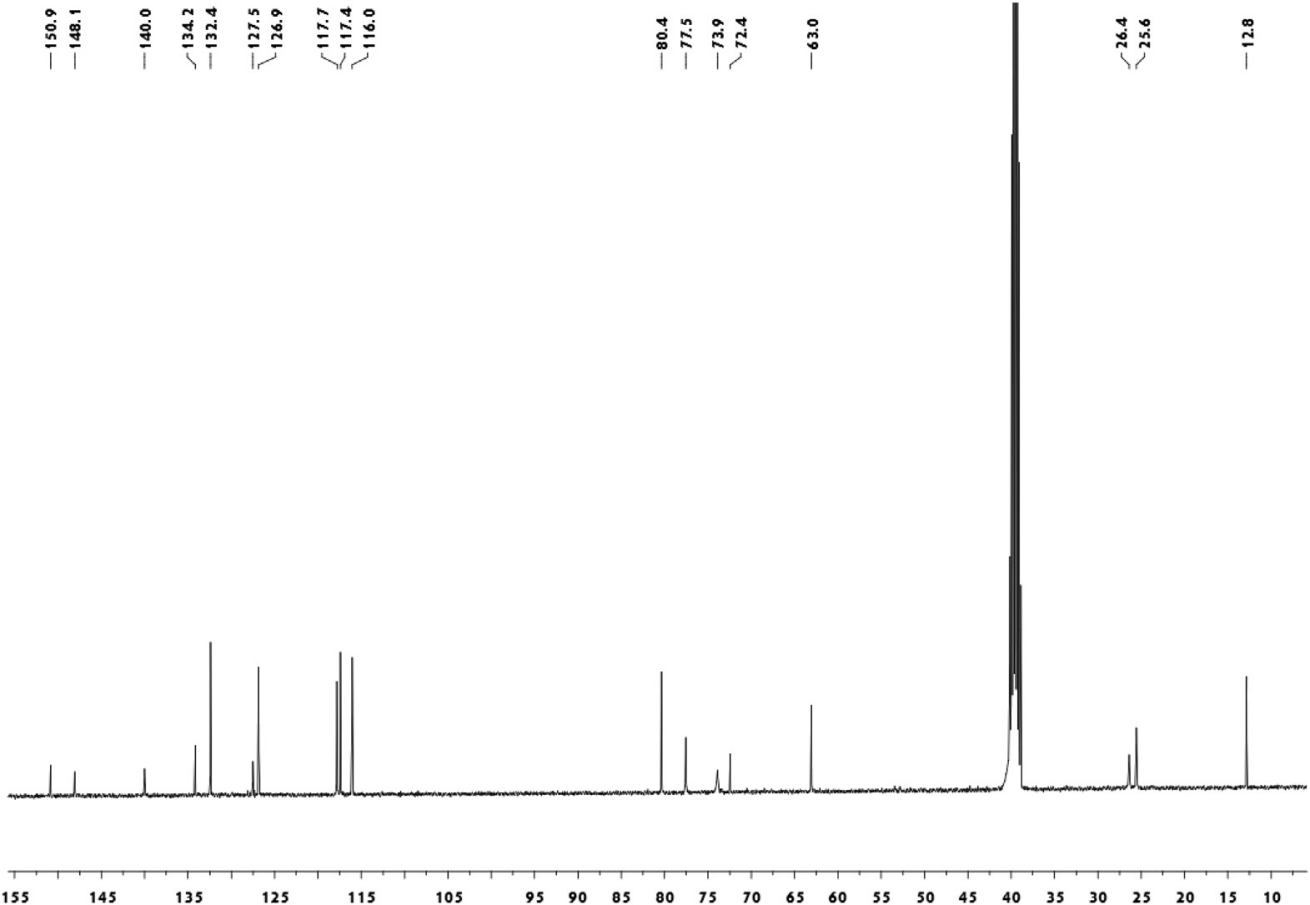
^13^C-NMR spectrum of meroterpenoid **2**.

Colorless crystals (MeOH—CH_2_Cl_2_); [α]_D_^20^ − 513.4 (c 0.15, CH_2_Cl_2_); ^1^H and ^13^C NMR spectrum see Fig. 1, Fig. 2.

#### 2.3.2 Meroterpenoid **5**

**Fig. 3:**
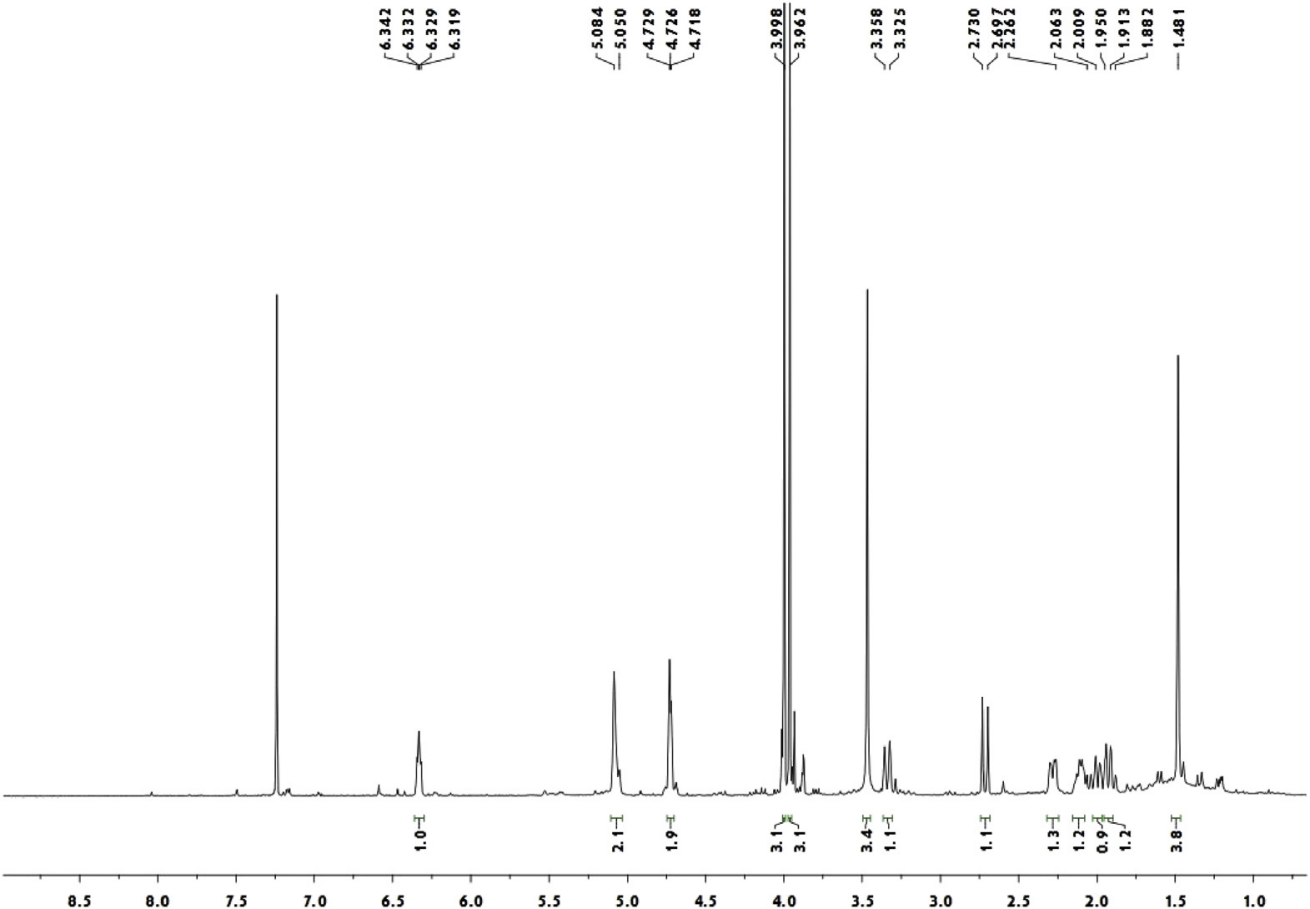
^1^H-NMR spectrum of meroterpenoid **5**.

**Fig. 4:**
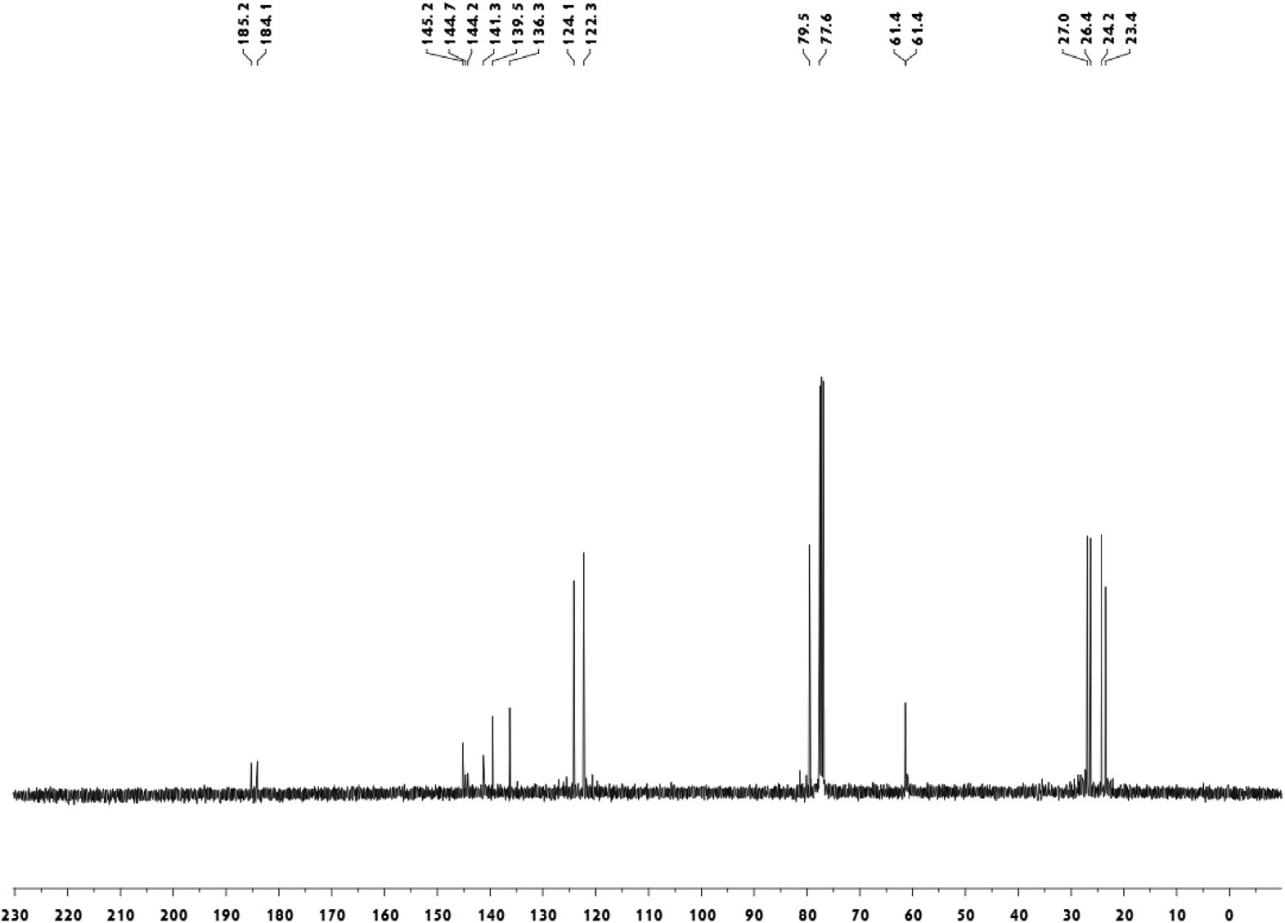
^13^C-NMR spectrum of meroterpenoid **5**.

Red crystal (MeOH—CH_2_Cl_2_); [α]_D_^20^ + 134.3 (c 0.10, MeOH); ^1^H and ^13^C NMR spectrum see Fig. 3, Fig. 4.

#### 2.3.3 Meroterpenoid **6**

**Fig. 5:**
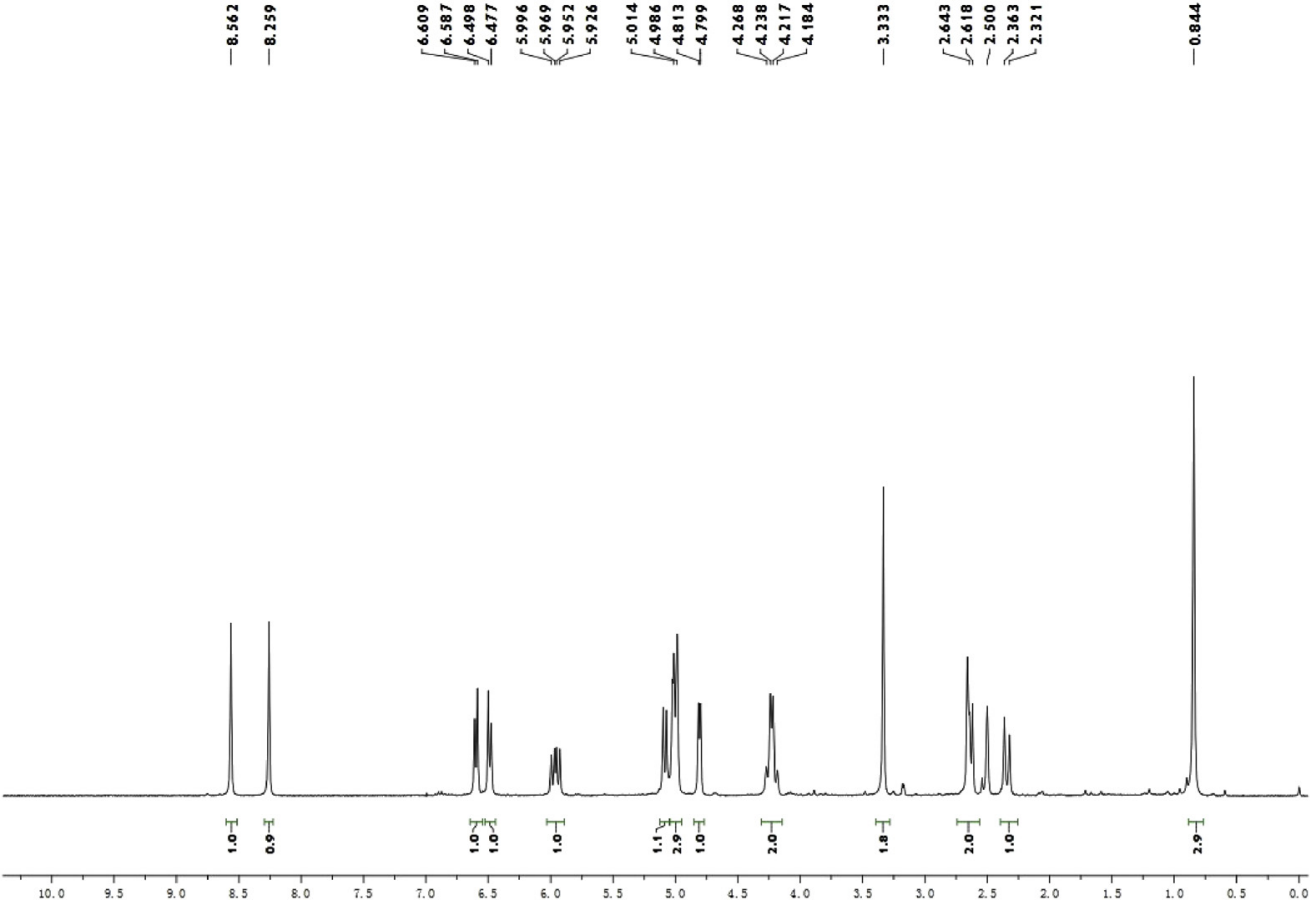
^1^H-NMR spectrum of meroterpenoid **6**.

**Fig. 6:**
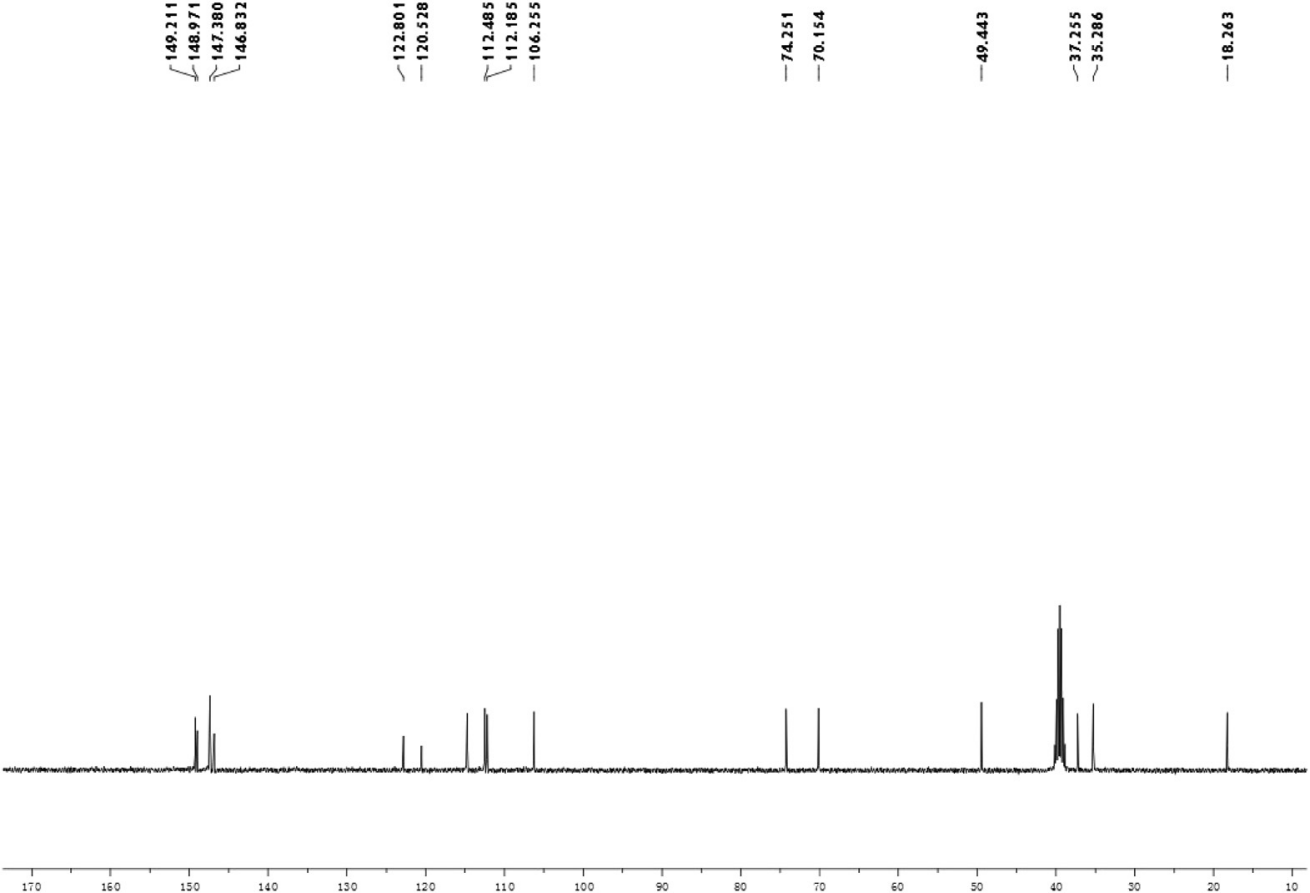
^13^C-NMR spectrum of meroterpenoid **6**.

White amorphous powder; [α]_D_^20^ − 21.4 (c 0.11, MeOH); ^1^H and ^13^C NMR spectrum see Fig. 5, Fig. 6.

#### 2.3.4 Meroterpenoid **7**

**Fig. 7:**
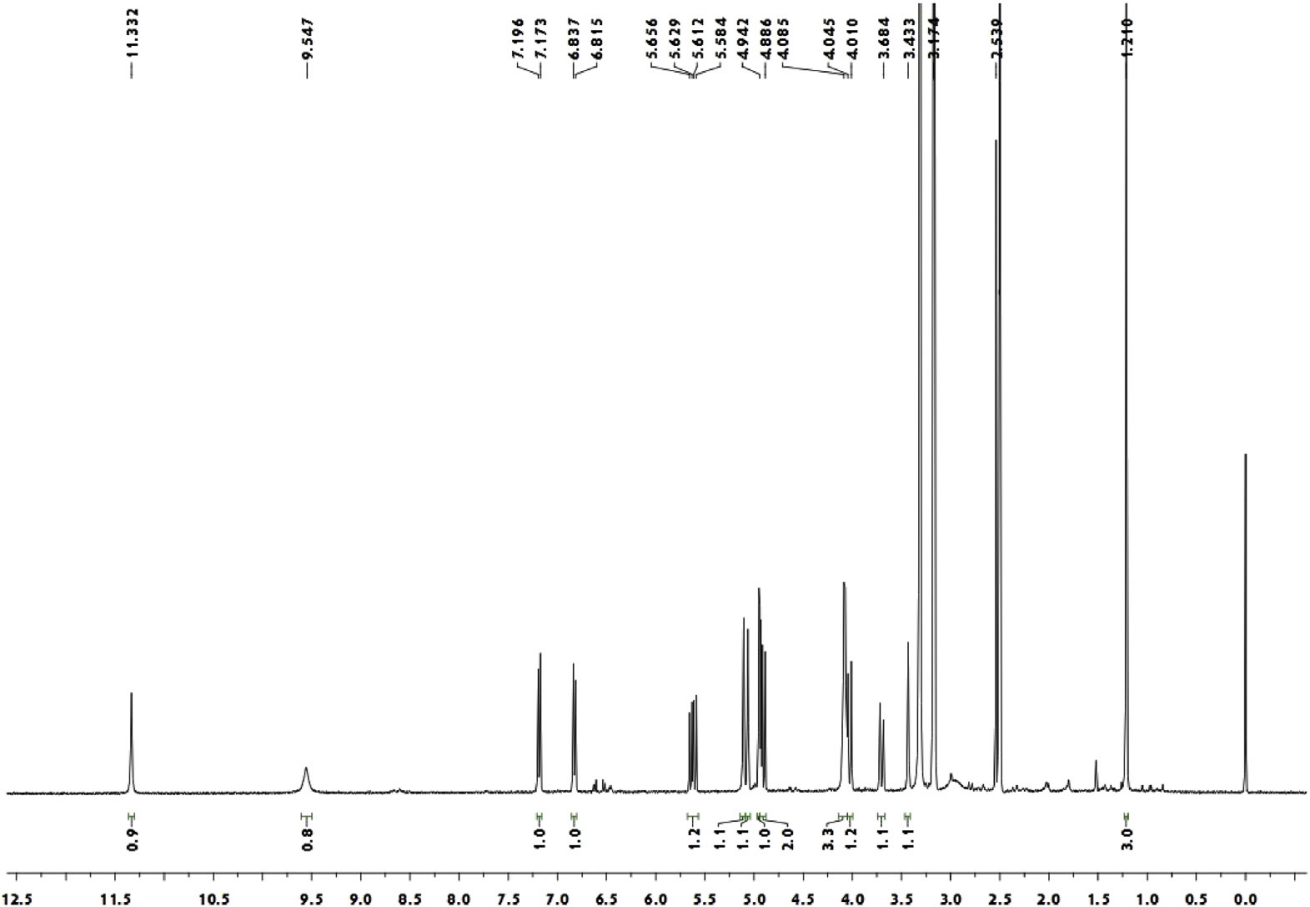
^1^H-NMR spectrum of meroterpenoid **7**.

**Fig. 8:**
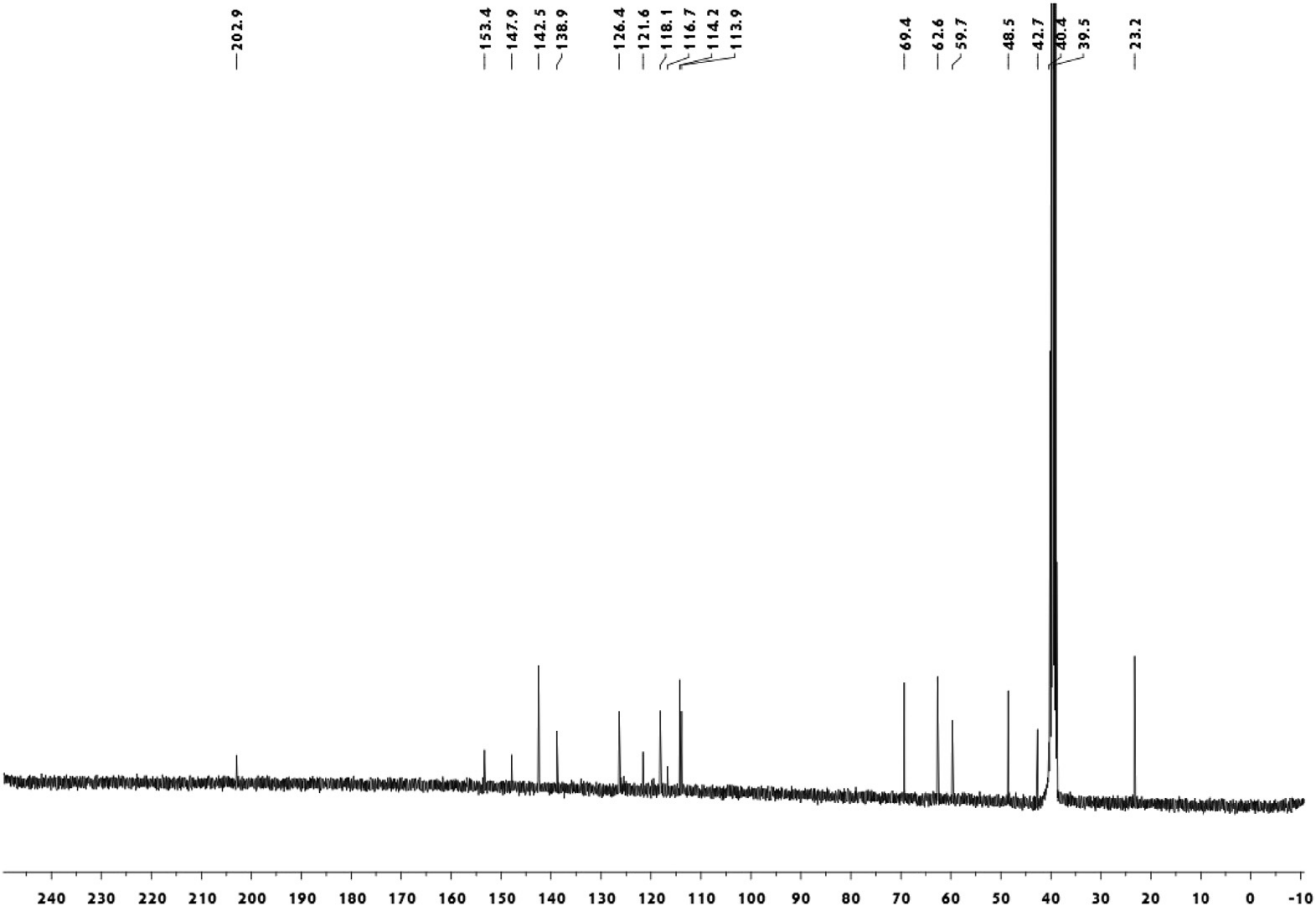
^13^C-NMR spectrum of meroterpenoid **7**.

Yellow amorphous powder; [α]_D_^20^ + 196.7 (c 0.10, MeOH); ^1^H NMR and ^13^C NMR spectroscopic data of meroterpenoid **7** see Fig. 7, Fig. 8.

#### 2.3.5 Meroterpenoid **8**

**Fig. 9:**
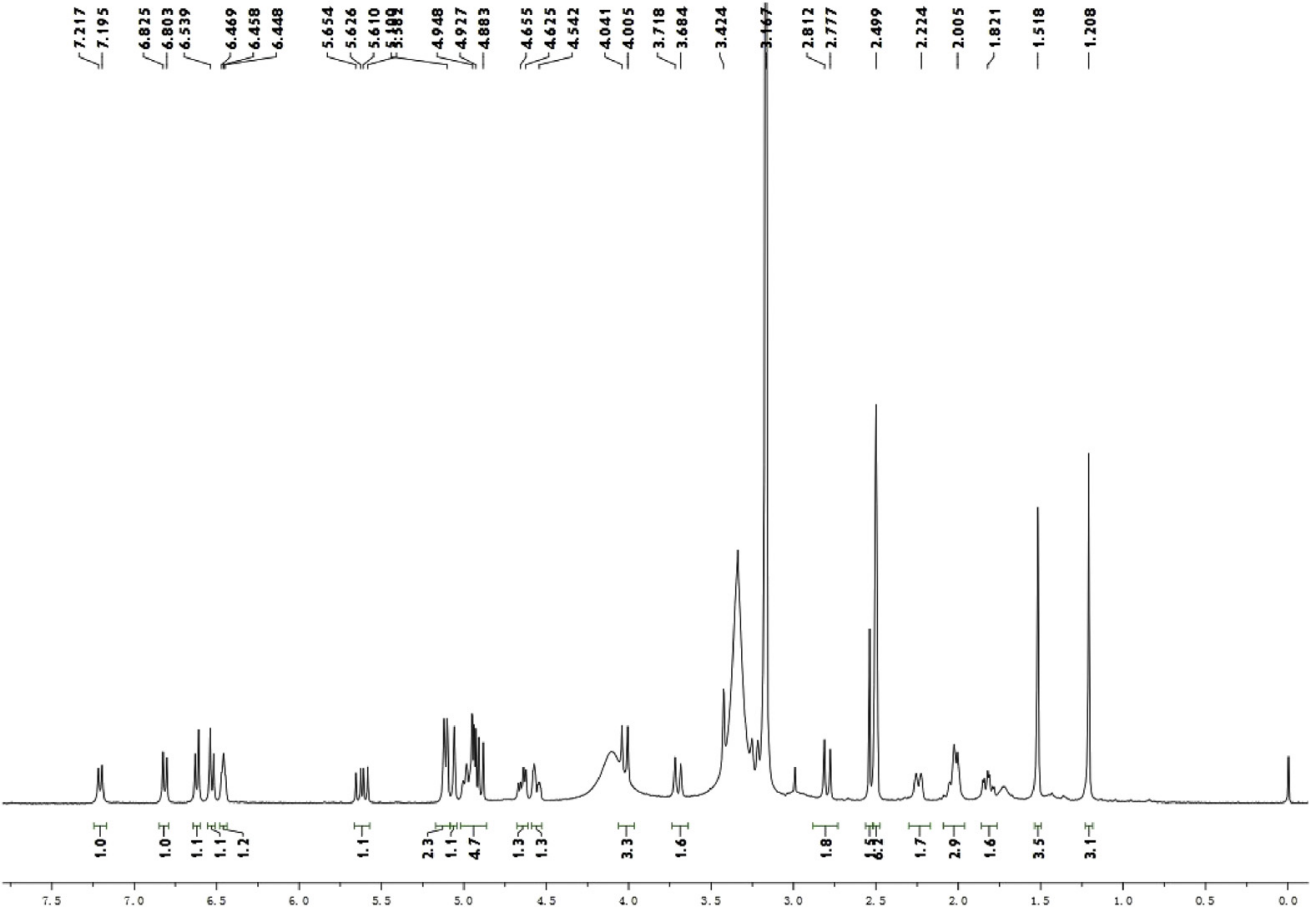
^1^H-NMR spectrum of meroterpenoid **8**.

**Fig. 10:**
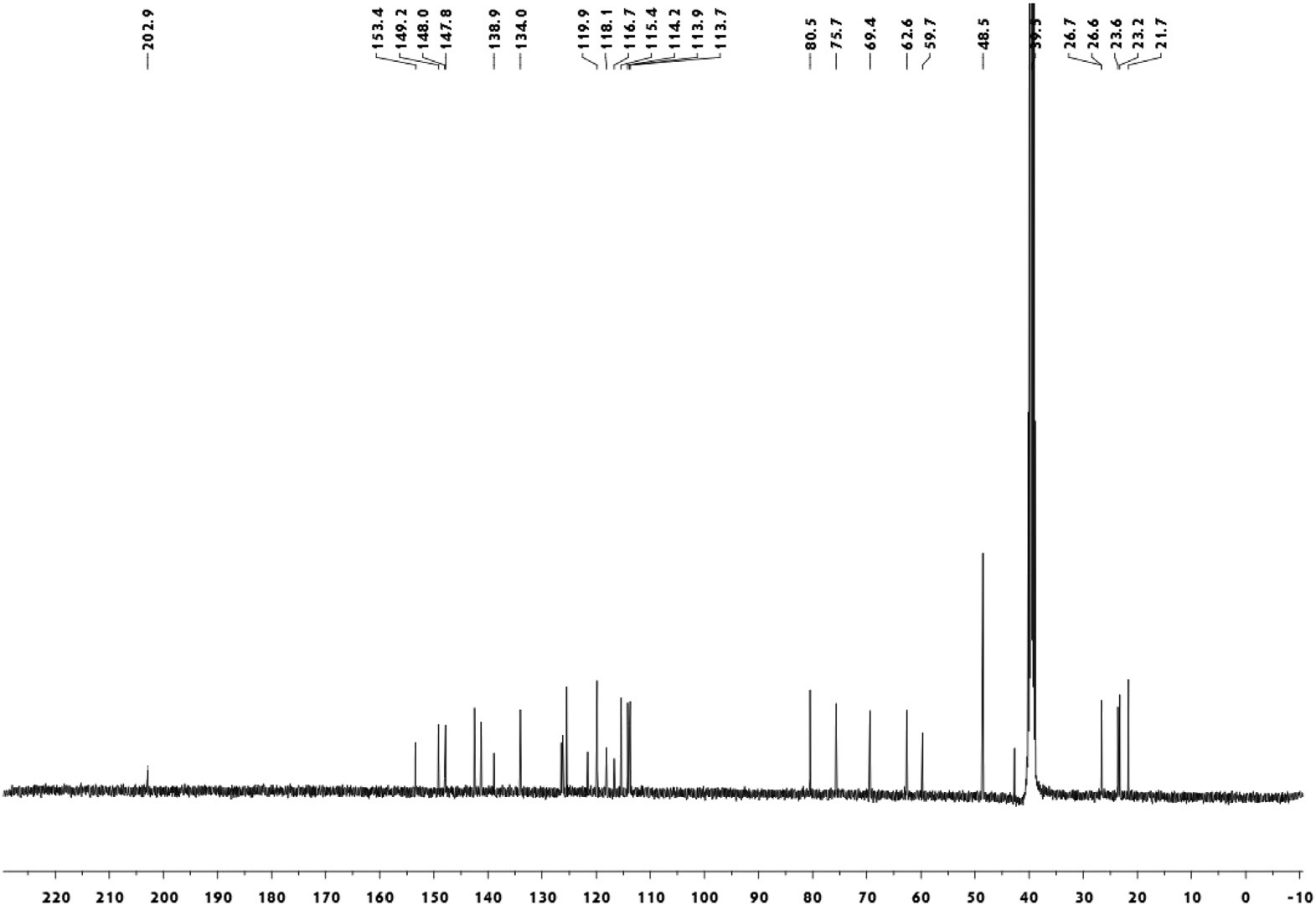
^13^C-NMR spectrum of meroterpenoid **8**.

Red amorphous powder; [α]_D_^20^ + 432.1 (c 0.10, MeOH); ^1^H NMR and ^13^C NMR spectroscopic data of meroterpenoid **8** see Fig. 9, Fig. 10.

#### 2.3.6 Meroterpenoid **9**

**Fig. 11:**
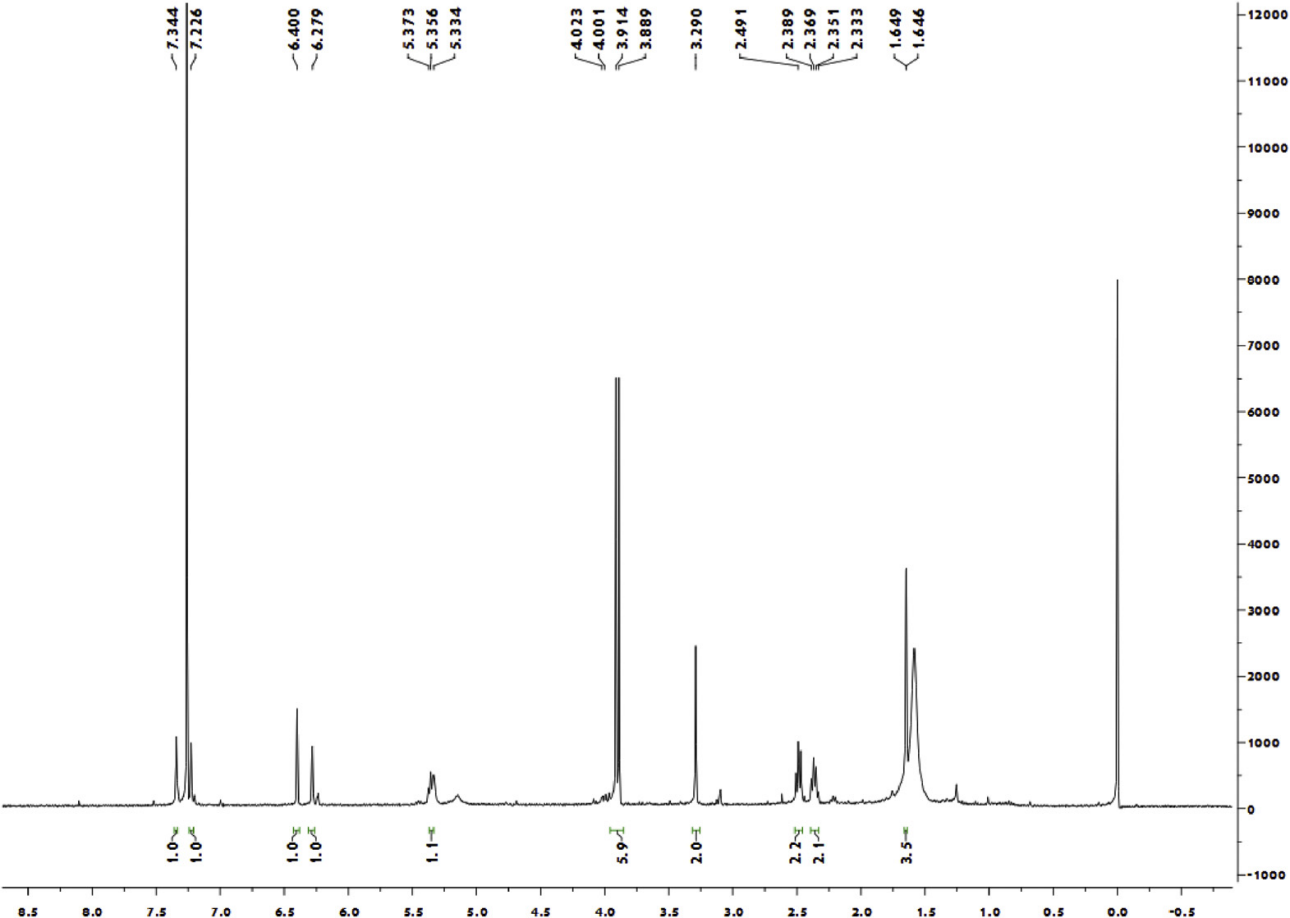
^1^H-NMR spectrum of meroterpenoid **9**.

**Fig. 12:**
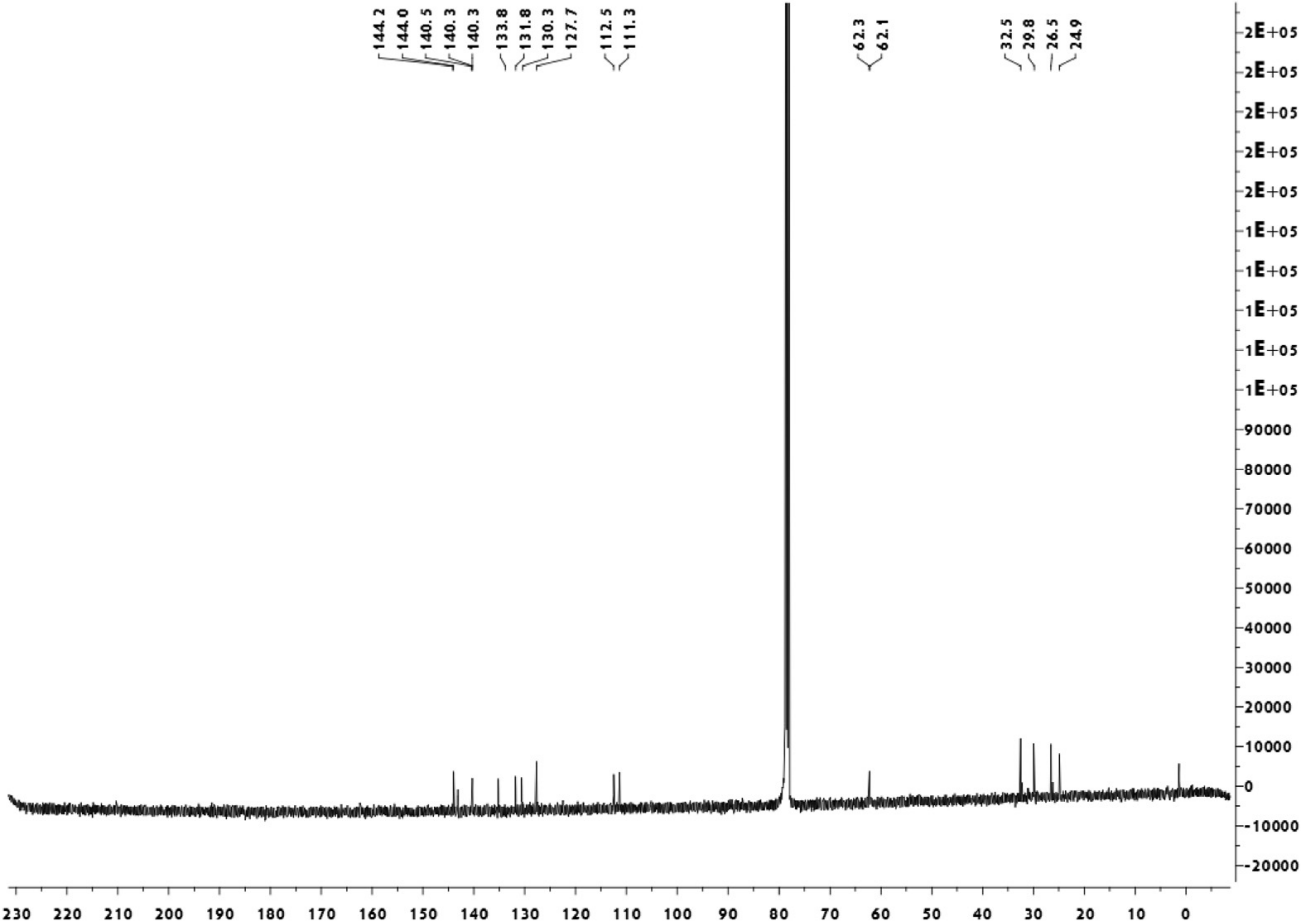
^13^C-NMR spectrum of meroterpenoid **9**.

Red amorphous powder; ^1^H NMR and ^13^C NMR spectroscopic data of meroterpenoid **9** see Fig. 11, Fig. 12.
